# Transcriptional Regulation of Sphingosine Kinase 1

**DOI:** 10.3390/cells9112437

**Published:** 2020-11-08

**Authors:** Joseph Bonica, Cungui Mao, Lina M. Obeid, Yusuf A. Hannun

**Affiliations:** 1 Department of Pharmacology, Stony Brook University, Stony Brook, NY 11794, USA; joseph.bonica@stonybrook.edu (J.B.); lina.obeid@stonybrookmedicine.edu (L.M.O.); 2 Department of Medicine, Stony Brook University, Stony Brook, NY 11794, USA; cungui.mao@stonybrookmedicine.edu; 3 Cancer Center, Stony Brook University, Stony Brook, NY 11794, USA

**Keywords:** sphingosine kinase 1, SK1, microRNA, transcription factor, hypoxia, long non-coding RNA

## Abstract

Once thought to be primarily structural in nature, sphingolipids have become increasingly appreciated as second messengers in a wide array of signaling pathways. Sphingosine kinase 1, or SK1, is one of two sphingosine kinases that phosphorylate sphingosine into sphingosine-1-phosphate (S1P). S1P is generally pro-inflammatory, pro-angiogenic, immunomodulatory, and pro-survival; therefore, high SK1 expression and activity have been associated with certain inflammatory diseases and cancer. It is thus important to develop an understanding of the regulation of SK1 expression and activity. In this review, we explore the current literature on SK1 transcriptional regulation, illustrating a complex system of transcription factors, cytokines, and even micro-RNAs (miRNAs) on the post transcriptional level.

## 1. Introduction

Sphingolipids are a class of cellular lipids that are involved in both maintaining cell structure and mediating cellular signaling processes. Sphingomyelins are important in cell membrane structure, while ceramides, sphingosine, and sphingosine-1-phosphate (S1P) are involved in cell signaling and are thus referred to as bioactive sphingolipids [1,2]. Ceramides can be produced by one of three pathways. The de novo pathway begins with the activity of serine-palmitoyl transferase to eventually generate ceramide. The hydrolytic pathway generates ceramide (and sphingosine) from the hydrolysis of complex sphingolipids. The salvage pathway involves re-generating ceramide and sphingolipids via salvage of sphingosine generated in the lysosome and then re-incorporated into ceramide. All three bioactive sphingolipids are known to have important signaling consequences in both healthy and diseased cell states. Ceramide and sphingosine are known to induce cell death, senescence, and/or differentiation and are generally antiproliferative [1,2]. S1P, on the other hand, is best known to increase cell survival, proliferation, angiogenesis, migration and invasion, and immune cell egress [2,3,4]. S1P typically signals through one of five G-protein coupled receptors, named S1PR1-5, although there is evidence it also signals intracellularly [5]. Due to the delicate and sometimes opposing nature of the signaling processes utilizing sphingolipids, the absolute and relative concentration of these lipids are tightly controlled [6]. As such, the enzymes involved in the metabolism of sphingolipids are also closely regulated, with their expression and activities modulated by the concentrations of lipids in the cell.

Sphingosine kinases catalyze the phosphorylation of sphingosine into S1P. There exist two known major isoforms of sphingosine kinase, products of the two distinct genes, sphingosine kinase 1 (SK1) and sphingosine kinase 2 (SK2). SK1 is typically localized to and more active at the plasma membrane [7,8], while SK2 is localized to the nucleus upon activation [9,10]. SK1 is the more closely studied of the two isozymes, and understanding its regulation and activity is important due to the pro-proliferative and pro-survival activities of its product S1P; this is especially true in cancer. SK1 is known to be highly upregulated in many cancers, such as breast cancer, colon cancer, head and neck cancer, and glioblastoma [11,12,13,14], and its upregulation is associated strongly with poor prognosis and increased cancer metastasis [15,16,17,18,19]. SK1 has also been shown to contribute to chemoresistance in cancer. In CML, for instance, higher expression of SK1 led to disruption in the ratio between C18 ceramide and S1P, contributing to imatinib resistance [20]. In melanoma, high SK1 expression is shown to contribute to resistance to immune checkpoint inhibitors [21]. Metabolically, SK1 drives the formation of S1P while also serving to clear sphingosine, and thus providing an exit from the sphingolipid metabolic network. As such, SK1 may also regulate the levels of ceramide and possibly other upstream sphingolipids. SK1 is thus considered a particularly key regulator of the levels of bioactive sphingolipids. Therefore, it is important to understand the mechanisms of SK1 regulation, to generate further study in cancer, and to identify potential drug targets.

SK1 regulation is well studied and has been discussed in several reviews [7,22,23]. However, it should be noted that work looking specifically at transcriptional regulation of the enzyme is somewhat limited. The gene for sphingosine kinase 1 is located on chromosome 17q25.2 [24]. Work on the rat sphk1 gene showed 6 exons, although 6 alternative first exons were also detected [25]. The human SPHK1 gene is 7 exons in length and 11,276 bp long.

The current body of work reveals that SK1 expression is regulated by several different transcription factors, indicating the enzyme’s importance in different signaling pathways. These pathways encompass such important conditions as neuronal growth, hypoxia, ischemia, and cancer. It is known that three primary isoforms of SK1 exist, namely, SK1a, SK1b, and SK1c [25,26,27]. The expression and distribution of these isoforms is partly governed by the methylation of CpG islands in the SK1 promoter [25]. At 384 amino acids, SK1a is the smallest isoform and the most highly expressed; SK1b is 398 amino acids long, and SK1c is 470 [27]. While not much is known about differences in activity or biological effects of these isoforms, there is evidence that SK1a and SK1b interact with different proteins in breast cancer [28]. Additionally, SK1b appears to be resistant to the SK1 inhibitor Ski in prostate cancer cells [29]. Otherwise, the three isoforms differ only in the size of their N-terminal regions [24]. There is also increasing evidence that SK1 regulation is partially governed by microRNAs, or miRNAs, and that dysregulation of miRNAs in cancer is responsible for higher expression of SK1. In this review, we discuss what is known about SK1 transcriptional and post-transcriptional regulation, what signaling pathways effect and are affected by SK1 regulation, and what further work needs to be done to fully understand regulation of this important enzyme.

## 2. Transcriptional Regulation-Sp1

**Figure 1:**
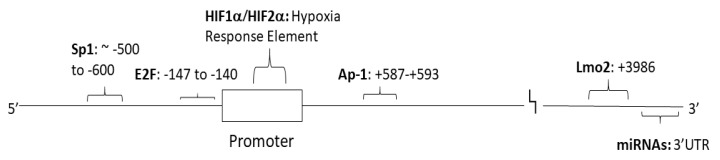
Location of transcription factor binding on the SK1 Gene. Approximate locations of *SPHK1* transcription factors in relation to the *SPHK1* promoter region. Transcription factors have been shown to bind both upstream and downstream of the promoter. E2F is known to be important in regulating SK1 in cancer, while Sp1 is associated with upregulating SK1 in cancer and in response to neuronal growth factors. In hypoxic conditions, HIF2a and Lmo2 were shown to upregulate SK1, especially in cancer models. AP-1 upregulates SK1 in response to the cytokine IL-1b. MicroRNAs (miRNAs) have been shown to bind to certain sequences in the 3’UTR of the *SPHK1* transcript.

Much work on SK1 transcriptional regulation implicates specificity protein 1, or Sp1, as an important transcription factor. In rat PC-12 cells, it was shown that neuronal growth factor (NGF) led to increased SK1 expression by increasing the expression of Sp1 [30]. In these cells, Sp1 was shown to interact specifically with exon 1d of the SK1 gene. Sp1 has also been shown to regulate SK1 in humans; for instance, Sp1 has been demonstrated to be packaged and transported in exosomes to upregulate SK1 in nearby cells, which protects from ischemia/perfusion injury [31]. Here, Sp1 is shown to interact with the region 0.5–0.6 kb upstream of the SK1 promoter (Figure 1). In a hepatocellular cancer model, blocking of Sp1 activity and expression with the known Sp1 inhibitor peretinoin decreased SK1 levels both in vitro and in vivo [32]. Interestingly, Sp1 is overexpressed in several cancers, such as breast, pancreatic, lung, glioma, and thyroid [33]. Many of these cancers also show upregulated SK1. Much like SK1, higher levels of Sp1 in these cancers are correlated with increased severity, stage, angiogenesis, and metastasis. This indicates an important relationship between SK1 and Sp1 and implies that increased Sk1 activity and expression can be used as a readout for conditions that increase Sp1 activity and/or expression.

## 3. Hypoxia and Ischemia

Due to S1P’s importance in angiogenesis, SK1 has long been studied as an important element in response to hypoxia. As oxygen levels decline, the body needs more blood vessels to move blood and oxygen quickly. This need for further angiogenesis serves to stimulate the upregulation of SK1 in hypoxic conditions. In endothelial cells, hypoxia is curiously shown to regulate SK1 but not SK2 [34], highlighting the former’s importance in angiogenesis. SK1 has a hypoxia response element (HRE) in its promoter (Figure 1), and as such, it responds to hypoxia sensitive transcription factors [34]. The regulation of SK1 in response to hypoxia has been demonstrated several times in living systems. For instance, SK1 has been shown to be upregulated in arteries after short periods of hypoxia [35]. Acute and chronic hypoxia have also been shown to upregulate SK1 in human pulmonary smooth muscle cells [36]. SK1 is also implicated in the regulation of two main hypoxia-induced transcription factors (HIFs), HIF1a and HIF2a [37].

Ischemia, which often leads to hypoxia, is known to regulate SK1, leading to both protective and deleterious effects on the involved system. For instance, SK1 was demonstrated to be substantially upregulated in a mouse stroke model, and its upregulation increased inflammatory response and poorer outcome [38]. A large increase in SK1 expression was similarly shown in the area of stroke lesion [39]. SK1 has also been shown to be upregulated in activated microglia, and to play a key role in the inflammatory response to cerebral ischemia-reperfusion (IR) [39,40,41,42]. Cerebral IR was shown in brain tissues to upregulate SK1, which in turn increased IL-17A expression in primary microglia [40]. The neuronal injury following cerebral IR in this model was reduced via administration of the SK1 inhibitor PF-543, cementing SK1’s role in driving inflammatory injury in this system. Further work demonstrated that SK1 affects IL-17 expression via upstream effects on TRAF2 expression and NFκB activation [41]. Crosstalk between SK1 and TLR2 has also been implicated in the inflammatory response to cerebral IR, as both were shown to be upregulated in microglia after cerebral IR [42]. In addition to PF-543, treatment of mice with fingolimod (a pro-drug functional antagonist of S1PR1) reduced hemorrhagic transformation and stroke injury [39]. These results suggest that targeting SK1 represents a potential treatment option for stroke and post-stroke injury, which have limited treatment options. Interestingly, SK1 upregulation induced by the anesthetic isoflurane is actually protective against intestinal injury in a renal ischemia model known to result in intestinal injury [43]. A separate renal IR injury model in mice showed that the increase in SK1 expression mediated the severity of the injury [44]. Upregulation of SK1 via exposure to conditioned media from mesenchymal stem cells in endothelial colony-forming cells potentiated the revascularization of endothelial colony-forming cells, considered an exciting means of treating infarct damage [45]. However, SK1 is also shown to be upregulated after myocardial infarction, and it contributes to dysfunctional cardiac remodeling and heart failure [46]. The specific transcription factors and regulatory pathways upstream of SK1 upregulation in ischemia do not appear to be widely studied; however, and future research in this area can improve our understanding of SK1’s various apparent roles in the ischemic response.

There is evidence that SK2 expression, on the other hand, offers protection against ischemic injury in a potentially compensatory manner. For instance, SK2 has been shown to be important in hypoxic preconditioning, and its activity and expression were required for the protective effects of such conditioning [47]. In cardiomyocytes, hypoxic preconditioning was responsible for increased expression of SK2, which was required to prevent apoptosis in ischemic conditions [48].

Hypoxia is an important response in cancer, due to often low oxygen conditions in the tumor; as such, hypoxic pathways are considered potential therapeutic targets in cancer [49]. SK1 is known to have several roles in cancer-induced hypoxia. Interestingly, most evidence points to HIF2alpha and not HIF1alpha as the primary regulator of SK1 in hypoxia [50,51]. In the U87MG glioma cell line, knockdown of HIF2a but not HIF1a led to downregulation of SK1 [50]. Transfection of cells with the SK1 promoter, however, led to upregulation of SK1 in response to CoCl2 treatment to simulate hypoxia, and several HRE elements were identified in the SK1 promoter [50]. HIF2a has also been associated with SK1 upregulation in clear cell renal carcinoma (ccRC) cells, where again knockdown of HIF2a led to reduced SK1 expression [51].There also exists evidence of a feedback loop between expression of HIF2a and SK1 in certain systems; a different model of ccRC from the one above seems to implicate SK1 in HIF2a regulation [52]. Another transcription factor important in angiogenesis, Lim domain only 2 transcription factor (Lmo2), has been shown to directly upregulate SK1 by binding to a sequence +3986 bases from the promoter [53]. This demonstrates the importance of SK1 in the hypoxic response, and especially the hypoxic cancer response.

## 4. Cytokines

SK1 and S1P are known to be involved in several inflammatory signaling pathways [23], and SK1 expression and activity are themselves regulated by several cytokines. In glioblastoma, it was found that SK1 is upregulated via the cytokine IL-1 through a JNK/c-Jun dependent pathway [26]. Since additional data show that high expression of SK1 correlates negatively with glioblastoma prognosis [17], understanding the mechanism of SK1 upregulation in response to cytokines is vital to understanding the disease severity. Interestingly, IL-1B only seems to upregulate the SK1a and SK1c isoforms in this system, with little effect on SK1b. Here, SK1 was shown to be upregulated by the binding of transcription factor AP-1 to the first intron at +587 to +593 [26].

In leukemia macrophage THP-1 cells, LPS initiated toll-like receptor 4 signaling stimulated SK1 expression, which led to the accumulation of IL-6 [54]. While evidence exists for SK1’s involvement in inflammatory pathways, evidence for cytokines themselves, leading to SK1 upregulation continues to be somewhat limited. It should be noted though, that cytokines have been also shown to activate SK1 through non-transcriptional mechanisms TNFα, for instance, is known to increase SK1 activity by inducing the phosphorylation of Ser 225, necessary for the trafficking of SK1 to the plasma membrane [55]. SK1 has also been shown to be activated by tumor necrosis factor receptor-associated factor 2 (TRAF2) via direct binding to the protein [56].

Transforming growth factor-B (TGF-B) signaling is known to upregulate and activate SK1, and SK1 activity has been shown to be important in many TGF-B dependent pathways. Treatment of fibroblasts with TGF-B led to increased SK1 expression, which was required for TIMP-1 upregulation [57,58]. TGF-B induced upregulation of SK1 has been shown to be important in coronary artery disease and liver fibrosis [58,59]. In breast cancer, TGF-B driven SK1 upregulation has been implicated in bone metastasis [60]. Despite the apparent link between the well-studied TGF-B signaling pathway and SK1 upregulation, the exact transcription factors and regulatory elements governing SK1’s response to TGF-B are currently not well established.

## 5. E2F and Long Non-Coding RNAs

In certain cancers, there is evidence of SK1 expression being under the control of transcription factors known as E2F transcription factors. This family of eight transcription factors is known to regulate proliferation, apoptosis, and stress responses [61]. Increased expression of E2Fs result in oncogenic activity in several cancers [62]. In head and neck squamous cell carcinoma, SK1 expression was shown to be downstream of E2F7, which is a direct transcription factor of SK1 [63]. In this cancer, E2F7-driven upregulation of SK1 is associated with anthracycline resistance; inhibition of E2F7 activity reduced SK1 levels and sensitized tumor cells to anthracycline chemotherapies. However, not all E2F family transcription factors regulate SK1 expression through direct actions on its promoter. For instance, the transcription factor E2F1 has been shown in liver carcinoma to regulate SK1 via the long non-coding RNA called HULC, or highly upregulated in liver cancer [64]. The connection between E2F transcription factors and SK1 regulation, especially in cancer, is one that merits further investigation based on these intriguing data.

Long non-coding RNAs, or lncRNAs, are RNA molecules of at least 200 nucleotides in length that do not code for any proteins [65]. While this type of RNA is incompletely understood, it is believed that they are largely regulatory in nature and known to be involved in epigenetic regulation [65,66]. While lncRNA’s role in disease is not completely understood, there exists compelling evidence that they are dysregulated in certain cancers [67]. In the case discussed above, the lncRNA HULC sequesters micro-RNA 107, a miRNA targeting E2F1; this preserves E2F1 and allows it to bind to the promoter [68]. As shown in Figure 1, E2F1 binds to the SK1 promoter at −147 to −140, stimulating expression. HULC’s activity in maintaining high SK1 levels is not limited to the liver, however, it has also been shown to upregulate SK1 in non-small cell lung carcinoma [69]. In that case, higher levels of SK1 prevent apoptosis and stimulate proliferation in the cancer cells.

Occasionally, lncRNAs are not transcribed from intergenic regions but from the antisense strand of a gene itself [66,67,68]. The SK1 gene, *SPHK1*, has its own antisense lncRNA called Khps1, of which several different subtypes exist [69]. Khps1 has been shown in rats to govern CpG island methylation of the *Sphk1* gene, and thus governing tissue differential expression of SK1 [69]. Khps1 has also been found to regulate SK1 by directly associating with histones and altering their structure [70]. However, work on the disease-specific effects of Khps1 on SK1 expression remains limited.

## 6. MicroRNAs

A growing body of work implicates microRNAs, or miRNAs, in SK1 regulation in both healthy and diseased states. MiRNAs are short oligonucleotides (typically ~22 nt in length), which degrade mRNAs by binding to their 3’untranslated region (3’UTR), targeting them for processing by the RISC [71]. Much of this regulation is relevant to cancer, where miRNAs are found to be frequently dysregulated. MiRNA dysregulation has, in fact, been linked to several diverse types of cancer [72]. There is also evidence of separate dysregulation of miRNA in the tumor microenvironment [73].

Several different miRNAs have been implicated in SK1 regulation, especially in cancer (Table 1). MiR-124, for instance, has been shown to regulate SK1 expression in a variety of cancers, with implications in invasion and metastasis, proliferation, and tumor formation. The 3’-UTR binding site of miR-124 in SK1 has been defined. Typically, miR-124 is downregulated in cancer, which leads to increased expression of SK1 and associated biology. In ovarian cancer, for instance, miR-124 overexpression in cell lines was shown to lead to SK1 degradation, which leads to reduced tumor invasion and migration [74]. This loss in invasive potential was restored with overexpression of SK1 in the cells. It has also been demonstrated that ovarian cancer-associated fibroblasts revert to normal fibroblasts when exposed to exosomes containing miR-124, and this is mediated via the degradation of SK1 in the CAFs [75]. MiR-124 also regulates SK1 in gastric cancer, leading to reduced proliferation and tumorigenicity [76]. MiR-124 has been shown to directly target SK1 in an osteosarcoma model, affecting proliferation, invasion, and matrix metalloprotease expression [77]. Outside of cancer, miR-124 promotes cell death in myocardial infarction by downregulating SK1 [78].

MiR-506 has also been established as an SK1 regulator in several different systems. In hepatocellular carcinoma, for instance, MiR-506 downregulated SK1 and inhibited angiogenesis [79]. This work also established a negative correlation between MiR-506 expression and SK1 expression in hepatocellular carcinoma tissues. MiR-506 is also downregulated in pancreatic cancer, and restoring its expression disrupts the SK1/AKT/NFkB axis [80]. This change both enhanced cancer cell death and increased sensitivity to gemcitabine, a chemotherapeutic commonly used in the treatment of pancreatic cancer. In osteosarcoma, a close relative of miR-506 called miR-506-3p inhibits SK1, leading to reduced invasion. Interestingly, the expression of this miRNA even causes the cells to lose metastatic potential via mesenchymal-to-epithelial transition [81].

While much work has focused on those two miRNAs in regulating SK1 expression, evidence has linked other miRNAs to SK1 regulation as well. MiR125b, for instance, has been shown to regulate SK1 expression in bladder cancer [82] and Alzheimer’s disease [68]. In the former case, overexpression of miR-125b reduced SK1 levels and proliferation [82]; in the latter case, expression of miR-125b was correlated to more severe disease, as SK1 was downregulated and cell death increased. In bladder cancer, the miRNA-613 is downregulated, leading to upregulation of SK1 and increased proliferation, migration, and EMT [83]. In colon cancer, miR-659-3p reduced SK1 and sensitized them to cisplatin [84], while in K562 leukemia cells, miR-659-3p reduced proliferation by targeting SK1 [68]. Hypoxic conditions downregulated expression of miR-1-3p in pulmonary smooth muscle cells, which led to upregulation of SK1 [85].

## 7. Discussion

Sphingosine kinase 1, or SK1, is a key enzyme in the sphingolipid pathway, as it converts the pro-death and pro-senescence lipid sphingosine into the pro-survival S1P. The dysregulation of SK1 has been associated with severity in several diseases, especially cancer. Higher expression of SK1 in several types of cancer is associated with poor survival and increased disease severity. High expression of SK1 increases cancer severity by driving proliferation, angiogenesis, metastasis, and chemoresistance through increased production of S1P.

While regulation of SK1 via post-translational modifications such as phosphorylation and proteolysis is well known, the means for transcriptional regulation of SK1 are somewhat less established. It is known that the transcription factor Sp1 upregulates SK1 under certain conditions, which is important in neuronal growth and possibly cancer. Hypoxia is also known to be vital to SK1 upregulation, with HIF2a being the primary transcriptional factor of SK1 in this case. In ischemia, upregulation of SK1 seems to have complex effects on recovery after injury, with the enzyme correlating with better or worse prognosis depending on the system. Despite these varying effects in ischemia, the transcription factors regulating SK1 expression in response to ischemia are not well defined. Further study of the transcriptional regulation of SK1 in ischemia can help broaden understanding of the development of ischemic injury and help establish SK1 as a drug target or possible upstream drug targets in this system. Cytokine signaling has also been shown to regulate SK1 expression, although again, the transcription factors governing this are not well established.

In cancer, the E2F family of transcription factors has recently been demonstrated to affect SK1 expression, which in turn was shown to improve chemoresistance and angiogenesis in tumors. Further work elucidating the connection between E2F transcription factors and SK1 in cancer would go a long way towards understanding the mechanism of these transcription factors in regulating disease. E2Fs have been shown to sometimes regulate SK1 expression via microRNAs, or miRNAs. Indeed, several different miRNAs have been found to regulate SK1 expression, and many of these miRNAs are downregulated in cancer.

Transcriptional upregulation of SK1 in several different diseases makes it an attractive therapeutic target. Some work has been done exploring the effect of small molecule inhibitors of known SK1 transcription factors on certain diseases. For instance, the pan-E2F inhibitor HLM006474 was shown to induce cell death in models of metastatic melanoma and lung cancer [86,87]. However, no work has been done to assess how disruption of SK1 expression may be related to these effects. The acyclic retinoid peretinoin has been shown to prevent carcinogenesis in liver fibroblasts by downregulating SK1 via Sp1 inhibition [32]. However, since transcription factors typically regulate the expression of several genes and since the effects of SK1 in several systems are well established, targeting of SK1 and the S1P pathway is more likely to be an effective treatment option. Experimentally, treatment with both the SK1 inhibitor PF-543 and the S1PR1antagonist fingolimod seemed to alleviate neuronal injury. Several in vivo studies have been conducted using various SK1 inhibitors, probing their effects on diseases such as asthma, sickle-cell anemia, multiple sclerosis, myocardial infarction, arthritis, and several cancers [88].

However, few clinical trials of SK1 inhibitors have been conducted. One such trial, looking at the putative SK1 inhibitor safingol in conjunction with cisplatin in solid tumors, showed that the inhibitor was well tolerated in patients [89]. Despite these safety data, little subsequent work has been done to measure the efficacy of SK1 inhibitors in human patients. In fact, a search of clinical trials occurring currently in the United States on clinicaltrials.gov reveals only 8 clinical trials, and they are mostly probing inhibitors of SK2 rather than SK1. Such work would be especially welcome in cancer, where SK1’s role is well established and where there remains a substantial need for targeted therapies. Further investigation should also be done on the role of SK1 in ischemia injury, as the initial results appear promising, and there is an enormous lack of pharmacological options for treatment. 

While considerable efforts have been applied to understanding SK1 regulation post-translationally, many elements of SK1 transcriptional regulation remain poorly understood. Some direct regulation via transcription factors has already been established, and we should examine SK1 levels in conditions where it is known that one of these transcription factors is more active. Post-transcriptional regulation also continues to be studied, and research into SK1 regulation via miRNAs is growing particularly rapidly. As we further elucidate just how miRNAs are regulated and effect certain disease states, so should we further study how miRNA downregulation of SK1 contributes to the disease state.

## Figures and Tables

**Table 1:** microRNAs Known to Downregulate SK1.

MicroRNA	Disease Association	Effects Due to SK1 Downregulation
miR-124	Ovarian Cancer [74]; gastric cancer [76], osteosarcoma [75,77], myocardial infarction [78]	Reduced migration and invasion [74,75]; reduced fibroblast cancer association [76], cell death [78]
miR-506	Hepatocellular carcinoma [79]; pancreatic cancer [80]; osteosarcoma [81].	Reduced angiogenesis [79]; enhanced chemosensitivity [80], mesenchymal-to-epithelial transition [81]
miR-125b	Bladder cancer [82], Alzheimer’s disease [68].	Reduced proliferation [82]; increased cell death and disease severity [68]
miR-613	Bladder Cancer [83]	Reduced proliferation, migration, EMT [83]
miR-659-3p	Colon cancer [84], leukemia [68].	Chemosensitivity [84], reduced proliferation [68]
miR-1-3p	Hypoxia in pulmonary smooth muscle cells [85]	Upregulation of Sk1 [85]
